# Repurposing drugs for the human dopamine transporter through WHALES descriptors-based virtual screening and bioactivity evaluation

**DOI:** 10.1016/j.jpha.2025.101368

**Published:** 2025-06-14

**Authors:** Ding Luo, Zhou Sha, Junli Mao, Jialing Liu, Yue Zhou, Haibo Wu, Weiwei Xue

**Affiliations:** a School of Pharmaceutical Sciences, Chongqing Key Laboratory of Natural Product Synthesis and Drug Research, Chongqing University, Chongqing, 401331, China; b School of Life Sciences, Chongqing University, Chongqing, 401331, China

**Keywords:** Dopamine transporter, Drug repurposing, Molecular descriptors, Virtual screening, Molecular dynamics simulation

## Abstract

Computational approaches, encompassing both physics-based and machine learning (ML) methodologies, have gained substantial traction in drug repurposing efforts targeting specific therapeutic entities. The human dopamine (DA) transporter (hDAT) is the primary therapeutic target of numerous psychiatric medications. However, traditional hDAT-targeting drugs, which interact with the primary binding site, encounter significant limitations, including addictive potential and stimulant effects. In this study, we propose an integrated workflow combining virtual screening based on weighted holistic atom localization and entity shape (WHALES) descriptors with *in vitro* experimental validation to repurpose novel hDAT-targeting drugs. Initially, WHALES descriptors facilitated a similarity search, employing four benztropine-like atypical inhibitors known to bind hDAT's allosteric site as templates. Consequently, from a compound library of 4,921 marketed and clinically tested drugs, we identified 27 candidate atypical inhibitors. Subsequently, ADMETlab was employed to predict the pharmacokinetic and toxicological properties of these candidates, while induced-fit docking (IFD) was performed to estimate their binding affinities. Six compounds were selected for *in vitro* assessments of neurotransmitter reuptake inhibitory activities. Among these, three exhibited significant inhibitory potency, with half maximal inhibitory concentration (IC_50_) values of 0.753 μM, 0.542 μM, and 1.210 μM, respectively. Finally, molecular dynamics (MD) simulations and end-point binding free energy analyses were conducted to elucidate and confirm the inhibitory mechanisms of the repurposed drugs against hDAT in its inward-open conformation. In conclusion, our study not only identifies promising active compounds as potential atypical inhibitors for novel therapeutic drug development targeting hDAT but also validates the effectiveness of our integrated computational and experimental workflow for drug repurposing.

## Highlights

• A workflow that integrates WHALES descriptors-based virtual screening and experimental validation has been developed for the repurposing of drugs against hDAT.
• From a library of 4921 drugs marketed or in clinical phases, three potential atypical hDAT inhibitors were identified, exhibiting IC_50_ values of 0.753, 0.542, and 1.210 μM, respectively.
• The binding modes of the repurposed drugs within the allosteric site of hDAT in inward-open conformation were elucidated by MD simulations and binding free energy analysis.

## Graphical abstract

**Figure:**
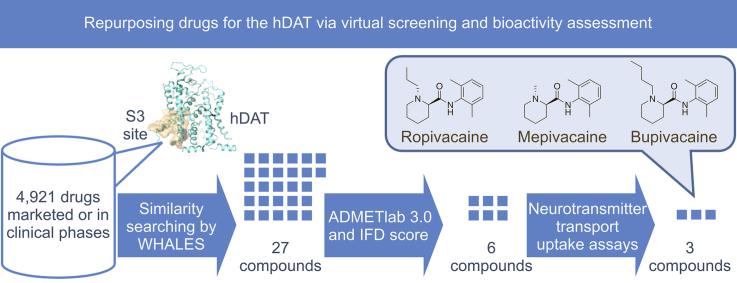

## 1 Introduction

The human dopamine (DA) transporter (hDAT) is a classic monoamine transporter (MAT) belonging to the neurotransmitter sodium symporter (NSS) family [1]. As a transmembrane protein, hDAT primarily controls extraneuronal DA concentrations in the brain, thereby modulating dopaminergic signaling within the central nervous system (CNS) [[2], [3], [4]]. Consequently, hDAT represents a principal pharmacological target for various psychiatric agents, notably including cocaine [5]. Although the mechanism of action (MOA) of hDAT has been extensively investigated [[6], [7], [8], [9]], most conventional hDAT-targeting drugs remain constrained by several critical limitations, such as structural similarity, singular binding-site interactions, and overlapping functional mechanisms [[10], [11], [12]].

**Fig. 1:**
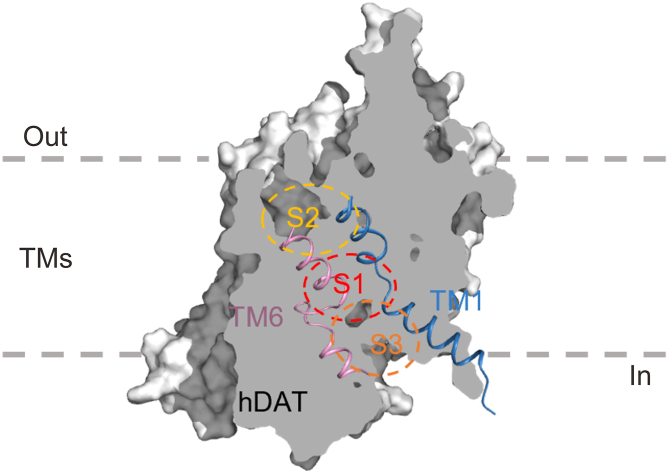
The three-dimensional (3D) structure and binding site distribution of human dopamine transporter (hDAT). The red circle denotes the orthosteric site, which serves as the binding site for dopamine and cocaine-like substrates. The yellow highlight identifies the S2 site, a region that exhibits structural heterogeneity in neurotransmitter sodium transporters, and is involved in allosteric modulation that regulates substrate uptake. The orange highlight marks the S3 site, located beneath the S1 site with partial overlap. This site corresponds to the binding regions for atypical inhibitors, such as benztropine (Protein Data Bank (PDB) ID: 8Y2E) and GBR12909 (PDB ID: 8H2F).

In recent years, atypical inhibitors and partial substrates have garnered attention as promising therapeutic alternatives due to their distinct pharmacological mechanisms [[13], [14], [15]]. These compounds induce and stabilize unique conformational changes in hDAT throughout its transport cycle, potentially resulting in diverse pharmacological outcomes [[16], [17], [18]]. This mechanism is similar to the ligand-specific pleiotropic functional properties that are inherent to G protein-coupled receptors (GPCRs) [19]. Such conformational modulations may lead to distinct behavioral and psychological responses, thereby underscoring the therapeutic potential of these novel compounds [15,20]. The concept of hDAT conformational specificity was initially proposed following observations that cocaine and benztropine elicit distinct effects on hDAT's outward-facing conformation [16]. Subsequent mutational analyses, including the Y335A mutation that stabilizes hDAT in its inward-facing conformation [17,21], revealed near-total abolishment of binding for cocaine-like compounds, whereas binding affinities of benztropine, JHW007, and *R*/*S*-modafinil were minimally impacted [20]. Additional evidence indicates that atypical inhibitors stabilize hDAT in occluded or inward-open states, translocate slowly, and effectively inhibit DA reuptake with minimal cocaine-like behavioral effects [17,22], a mechanism analogous to ibogaine's action on human serotonin transporter (hSERT) and hDAT [23,24]. Recently resolved cryo-electron microscopy (cryo-EM) structures have confirmed that the atypical inhibitor benztropine binds specifically to the S3 site of hDAT [8] (Fig. 1).

Recent advancements in machine learning (ML), particularly deep learning (DL), have substantially accelerated the identification of hit compounds in drug discovery [25,26]. ML models now enable the analysis of extensive chemical libraries, facilitate the accurate prediction of compound bioactivities, support drug repurposing efforts, and optimize drug-like properties with unprecedented efficacy [[27], [28], [29]]. However, the choice of feature extraction methods for compound representation is critical when employing ML approaches [[30], [31], [32]]. Typically, one-dimensional (1D) sequences (such as simplified molecular input line entry system (SMILES)), 2D, and 3D molecular structures have been employed as input data for feature extraction [33,34]. Prior studies have developed the novel weighted holistic atom localization and entity shape (WHALES) descriptors derived from 3D molecular structures [35], which have proven effective in identifying new modulators of the retinoid X receptor (RXR) [36]. These descriptors enhance ML model accuracy by encoding critical molecular features, including spatial, electronic, and physicochemical properties, essential for bioactivity prediction [37]. Furthermore, descriptor-based models have proven pivotal in virtual screening applications, such as similarity searches, enabling the efficient identification of compounds with high binding potential across diverse chemical spaces [36,38]. Collectively, these advancements have facilitated the discovery of novel hit compounds targeting specific proteins, significantly enhancing efficiency and precision during early-stage drug discovery [11,39].

**Fig. 2:**
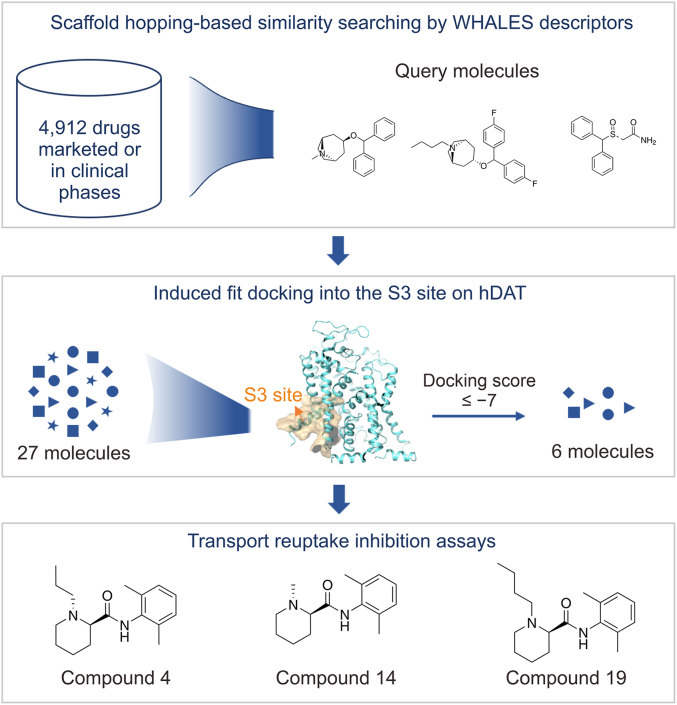
The workflow for the drug repurposing against human dopamine transporter (hDAT) through scaffold hopping-based virtual screening and bioactivity assessment. Firstly, a similarity search algorithm based on scaffold hopping, weighted holistic atom localization and entity shape (WHALES), was used. Benztropine, JHW007, and *R*/*S*-modafinil were utilized as query molecules to screen from a library of 4,921 compounds, yielding 27 molecules with high similarity. These 27 molecules were docked to the S3 site of hDAT through induced fit docking. Six molecules with reasonable docking scores and poses were selected for transport inhibition activity testing, and ultimately, three active molecules were identified.

In this study, we focus on benztropine-like atypical inhibitors of hDAT, which specifically target the S3 binding site located in the intracellular vestibule [15,40,41]. The integrated workflow depicted in Fig. 2 outlines our strategy for drug repurposing targeting hDAT, combining virtual screening based on WHALES descriptors with subsequent bioactivity validation. Initially, employing four benztropine-like atypical inhibitors known to bind to the S3 site as query templates, we conducted a similarity search and identified 27 structurally diverse scaffold-hopped molecules from a library consisting of marketed or clinically investigated drugs. The physicochemical properties and medicinal chemistry profiles of these candidate molecules were then assessed using ADMETlab 3.0 [42] to ensure their drug-likeness. Subsequently, binding affinities of the candidate compounds were evaluated via induced-fit docking (IFD). Six compounds with promising docking results were selected and purchased for further *in vitro* experimental validation to assess their inhibitory activities on neurotransmitter reuptake, resulting in the identification of three potential atypical inhibitors of hDAT. Finally, molecular dynamics (MD) simulations combined with end-point binding free energy calculations were performed to elucidate the binding modes and validate the inhibitory mechanisms of these three active compounds at the S3 site of hDAT.

## 2 Materials and methods

### 2.1 Data preparation

To repurpose drugs for hDAT, we initially identified four compounds, including benztropine, JHW007, and *R*/*S*-modafinil, which have been previously identified as atypical hDAT inhibitors. These compounds served as templates for conducting similarity searches. Subsequently, we selected the drug repurposing compound library (Product No.: L9200) from TargetMol as the screening repository. This library comprises 4,921 approved or clinically developed drugs, making them suitable candidates for drug screening applications [43]. All molecules were stored in 3D SDF format for subsequent similarity searches.

### 2.2 Similarity search based on WHALES descriptors

Traditionally, 1D or 2D molecular fingerprints, such as SMILES encoding or 2D fingerprint maps, have been employed for similarity searching [44,45]. These represent relatively simple structural depictions that, while containing sufficient information, allow for effective similarity-based virtual screening [46]. However, in our approach, to enhance molecular structure representation, we incorporated 3D molecular scaffold data into the similarity search framework. This method enables a more complete sampling of the molecular features and spatial arrangement, thereby improving accuracy in virtual screening. Therefore, we employed WHALES descriptors [36] to conduct similarity searches. WHALES offers a holistic molecular representation by integrating multidimensional information, including interatomic geometric distances, molecular shape, and atomic properties.

The process began with the calculation of the 3D structures of all molecules which were subsequently converted into WHALES descriptors using RDKit. This involved several steps. First, molecular conformations were subjected to energy minimization using the Merck Molecular Force Field (MMFF94) [47,48], followed by the calculation of Gasteiger-Marsili partial charges [49]. Based on the minimized conformations and partial charges, an atom-centered weighted covariance matrix was computed (Eq. (1)). *X*_*i*_ and *X*_*j*_ represent the 3D coordinates of the *i*th and *j*th atoms, respectively, and |δ_*i*_| denotes the absolute value of the partial charge of atom *i*.

(1) $$S_{w\left(j\right)}=\frac{\sum_{i=1}^{n}\left|\delta_{i}\right|\cdot \left(x_{i}-x_{j}\right){\left(x_{i}-x_{j}\right)}^{T}}{\sum_{i=1}^{n}\left|\delta_{i}\right|}$$

The atom-centered Mahalanobis (ACM) distance matrix was then calculated to further characterize the molecules (Eq. (2)).

(2) $$\text{ACM}\left(i,j\right)={\left(x_{i}-x_{j}\right)}^{{T\setminus \text{cdot}S}_{w\left(j\right)}^{-1}}\cdot \left(x_{i}-x_{j}\right)$$

Next, three essential atomic indices were determined: isolation (Isol.), remoteness (Rem.), and isolation-remoteness ratio (IR) (Eqs. (3), (4), (5))).

(3) $$\text{Isol}\left(j\right)=\text{mi}n_{i}\left(\text{ACM}\left(i,j\right)\right)\;i\neq j$$

(4) $$\text{Rem}\left(j\right)=\frac{\sum_{i=1}^{n}rACM\left(j,i\right)}{n-1}$$

(5) $$\text{IR}\left(j\right)=\frac{\text{Isol}\left(j\right)}{\text{Rem}\left(j\right)}$$

These indices provided both local and global information about the atoms within the molecule. To assemble the final WHALES descriptors, the maximum, minimum, and decile values of these indices were computed for each molecule. Since each index contributed 11 values, the complete WHALES descriptor for each molecule consisted of 33 values.

During the similarity search, the degree of similarity between the template molecule and the query molecule was quantified using the Euclidean distance between their respective WHALES descriptors (Eq. (6)). A smaller Euclidean distance indicated a higher degree of similarity between the molecules.

(6) $$D_{xy}=\sqrt{\sum_{j=1}^{p}{\left(x_{j}-y_{j}\right)}^{2}}$$

### 2.3 Prediction of drug properties by DL-based method ADMETlab 3.0

Drug property predictions were conducted using the DL-based ADMETlab 3.0 platform [42]. The method was chosen for its comprehensive 77 predictive models and its improved multi-task graph neural network (MT-GNN) structure [50], The DMPNN-Des framework used in version 3.0 combines Directed Message Passing Neural Networks (DMPNN) with RDKit 2D descriptors to create a detailed molecular embedding [51,52]. This process involved transforming each molecule into both a molecular graph and an RDKit 2D descriptor vector, using the DMPNN to learn atomic and bond features, aggregating these with 2D descriptors, and then applying a feed-forward neural network to predict drug properties.

We first obtained the standardized 3D structures of the 27 compounds by downloading them from PubChem [53] or drawing structures with ChemDraw and were optimized using LigPrep [54]. The optimized structures were compiled into a single SDF file, which was subsequently uploaded to the ADMETlab 3.0 server (https://admetlab3.scbdd.com/) for physicochemical and drug-like property predictions [42]. The prediction results were exported as a CSV file, from which Lipinski's Rule of Five data were manually extracted and presented in Table S1. All 27 compounds met the criteria for drug-likeness as outlined by Lipinski's rules.

### 2.4 IFD

Molecular docking is one of the efficacious structure-based computational methods to predict the interactions between ligands and targets at the atomic level [55]. Compared to rigid docking, IFD provides more reasonable docking conformations by incorporating the calculation of protein flexibility. In IFD, both the ligand and the receptor are considered flexible. Initially, the ligand approaches the receptor. As they interact, conformational changes occur in both the ligand and the receptor. The receptor adjusts its shape to accommodate the ligand better, while simultaneously adjusting its own conformation to fit into the receptor's binding site more precisely. This is different from the traditional lock-and-key model which assumes both receptor and ligand rigidity. During the course of this work, the crystal structure of the hDAT had not yet been resolved. We utilized the inward-facing DA-DAT complex structure from our previous study [7], which was characterized through homology modeling and Gaussian-accelerated MD (GaMD) simulations [56], to define the binding pocket S3. This pocket is located beneath the orthosteric site at the center of hDAT and partially overlaps with it (Fig. 1). Then, we utilized the IFD method for predicting the binding mode between ligands with the S3 pocket in hDAT. Prior to IFD, the compounds identified from scaffold hopping-based similarity search were energy optimized by LigPrep in Schrodinger software with OPLS4 force field [57], resulting in the generation of corresponding 3D conformations. Subsequently, the Epik algorithm [58] was employed for ionization handling at a pH value of 7.0 ± 2.0. The centroid of grid box on hDAT was defined by residues Gly75, Phe76, Asp79, Val328, Asp421, and Gly425 in the DA-DAT complex, and the docking size was set with a length ≤15 Å^3^. The side chains of residues within a 5 Å range around the optimized ligand were set up. No atoms were set as restrictions for hydrogen bonds or metals. The sampling of the ligand's ring conformation was enabled, and the energy window was set to 2.5 kcal/mol. In the IFD process, each ligand was set to generate 20 docking poses. The resulting complexes were evaluated and ranked based on binding energy by Prime [59] and Glide XP scoring [60].

### 2.5 Neurotransmitter transport uptake assays

DA uptake activity was measured using a Neurotransmitter Transporter Uptake Assay Kit (Molecular Devices, San Jose, CA, USA). To avoid the influence of other non-specific transporters, we chose the HEK-293T cell line that stably expressed hDAT, and it also has the advantages of rapid proliferation, easy cultivation, and high transfection efficiency. HEK-293T cell line was plated overnight at 6 × 10^4^ cells/well (96-well plate) under 37 °C, 5% CO_2_. 2-[4-(2-hydroxyethyl)-1-piperazinyl] ethanesulfonic acid (HEPES) buffer solution (150 mM NaCl, 5 mM KCl, 10 mM glucose, 2 mM CaCl_2_, 1 mM MgCl_2_, 10 mM HEPES, pH 7.4) was used to wash cells three times before the experiment. Cells in the experimental group were treated with six different compounds. After 15 min, the dye solution was added and the fluorescence intensity was measured for 30 min in the 520 nm range with excitation at 440 nm. The inhibition percentages were calculated using the end point measurements at 30 min. The four-parameter logistic nonlinear regression model was used to determine the half maximal inhibitory concentration (IC_50_) values.

### 2.6 MD simulation

In this work, six systems (Benztropine-DAT, JHW007-DAT, Modafinil-DAT, compounds 4 and 14, and 19-DAT) were prepared and subjected to MD simulations with a GPU-accelerate version of AMBER20 [61]. To resolve unfavorable contacts between solute molecules and solvent water in the system, energy minimization and equilibration simulations were conducted in three phases prior to the production run. First, energy minimization was performed by applying harmonic restraints to the lipid and solute atoms (force constant = 10 kcal/mol/Å^2^). The entire system was initially minimized for 10,000 steps, followed by a further 5,000 steps using the Steepest Descent algorithm and the conjugate gradient method. In the second phase, a two-step equilibration process was applied. The system was heated incrementally from −273.15 °C to −173.15 °C and then to 36.85 °C, with restraints on the protein and lipid maintained over 100 ps in the NVT ensemble. Subsequently, each system underwent 3 cycles of unconstrained NPT dynamics at 36.85 °C and 1 atm for 5 ns. Finally, a 100 ns MD production run was performed for each complex under the NPT ensemble at 36.85 °C and 1 atm using periodic boundary conditions. Temperature and pressure were regulated with a Langevin thermostat and a Monte Carlo barostat, respectively. Long-range electrostatic interactions were calculated with the particle-mesh Ewald (PME) method, using a cutoff distance of 10 Å. The SHAKE algorithm was employed to constrain all bonds, and the time step was set to 2.0 fs [62].

### 2.7 End-point binding free energy estimation

The molecular mechanics/generalized born surface area (MM/GBSA) method was utilized to calculate the binding free energy (Δ*G*_calc_) of these compounds to hDAT. The calculation was based on the following Eq. (7).

(7) $$\Delta G_{\text{calc}}=\Delta E_{\text{vdw}}+\Delta E_{\text{ele}}+\Delta G_{\text{pol}}+\Delta G_{\text{nonpol}}$$

For each term, Δ*E*_vdW_ refers to the van der Waals energies and Δ*E*_ele_ refers to the electrostatic interaction energies in the gas phase. Δ*G*_pol_ and Δ*G*_nonpol_ represent the polar and nonpolar solvation energies, respectively.

### 2.8 Molecular interaction fingerprint (MIF) analysis

The MIF between hDAT and different ligands were analyzed using 100 snapshots extracted from the simulation trajectory of each complex by ProLIF [63]. The interaction between ligand and protein over time was visualized by calculating the type of interaction between the ligand and residues within 10 Å in each frame, including hydrogen bond interactions, van der Waals force interactions, hydrophobic interactions, strong cation interactions, cation-π interactions, and π-stacking interactions.

## 3 Results and discussion

### 3.1 Potential hDAT inhibitors identified through scaffold hopping

Traditional drugs targeting the orthosteric site of hDAT exhibit significant structural similarities and are associated with severe clinical side effects, such as addiction [8]. Conversely, atypical inhibitors, including benztropine, JHW007, and *R*/*S*-modafinil, selectively bind the inward-open conformation of hDAT [15,16,22], thereby significantly reducing reinforcing effects and locomotor stimulant properties, particularly in humans [64]. Therefore, the rational design and discovery of novel hDAT inhibitors with structurally distinct scaffolds and unique inhibitory mechanisms has become increasingly important. To overcome these limitations, we selected four atypical inhibitors as templates for a WHALES descriptors-based similarity search aimed at repurposing drugs targeting the inward-open conformation of hDAT. The WHALES descriptors were initially developed to facilitate scaffold hopping by transferring structural and pharmacophore information from bioactive natural products to synthetic analogs [36].

**Fig. 3:**
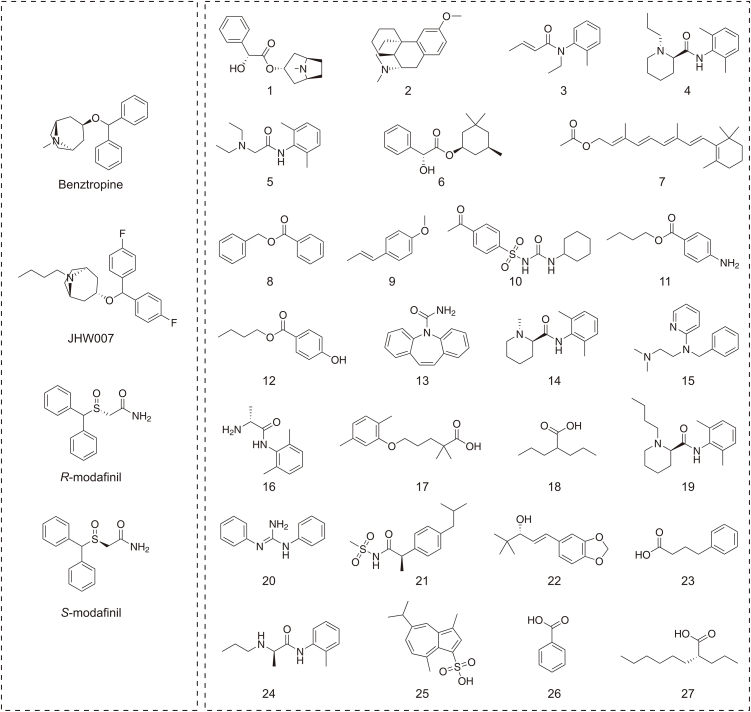
Four atypical inhibitors (left), considered to bind within the S3 pocket of human dopamine transporter (hDAT), have been shown to effectively obstruct hDAT-mediated substrate translocation. The inward-open conformation of hDAT, bound with benztropine, has been characterized by cryo-electron microscopy (cryo-EM). These four compounds were utilized as template molecules for scaffold-based similarity searching, leading to the identification of 27 structurally diverse compounds (right). The physicochemical properties of these compounds were subsequently validated using ADMETlab 3.0, confirming their suitability as potential drug candidates.

Through molecular scaffold similarity ranking, 27 potential hDAT inhibitors were identified from a library of 4,921 commercially available compounds, either marketed or in clinical development stages (Fig. 3). The pharmacokinetic and toxicity profiles of these candidates were further evaluated using the DL-based predictive model ADMETlab 3.0. The predicted drug-likeness data for these 27 molecules, based on Lipinski's Rule of Five, are summarized in Table S1, and all candidates demonstrated favorable pharmacokinetic properties. Among them, eight molecules exhibited blood-brain barrier (BBB) permeability coefficients between 0.9 and 1.0, indicating their suitability as CNS drugs. Furthermore, 26 molecules possessed logarithmic *n*-octanol/water distribution coefficients (log*P*) ranging between 1 and 5. The relative molecular weights (MWs) of all candidates were within 100–400 Da, 25 compounds had hydrogen bond acceptors ranging from 2 to 5, 25 molecules had hydrogen bond donors ranging from 0 to 2, and 23 molecules had topological polar surface area (TPSA) values between 20 and 90. Notably, the three active compounds identified in this study (compounds 4, 14, and 19) satisfied all these drug-likeness criteria.

Regarding potential toxicological profiles, previous research indicated that compound 4 (*Ropivacaine*) could induce mild CNS and cardiac toxicity, although it remains widely utilized for nerve block and postoperative analgesia [65]. Predictive analysis using ADMETlab suggested minimal hematotoxicity for compound 14 (*Mepivacaine*); however, rapid metabolism restricts its application to peripheral nerve blockade [66,67]. Similarly, compound 19 (*Bupivacaine*) has demonstrated mild CNS and cardiac toxicity, particularly under conditions of acidosis and hyperkalemia [[68], [69], [70]]. Despite these considerations, the side-effect profiles of these compounds are considered manageable and clinically acceptable.

### 3.2 The predicted binding modes and affinities of the repurposed drugs to hDAT

**Fig. 4:**
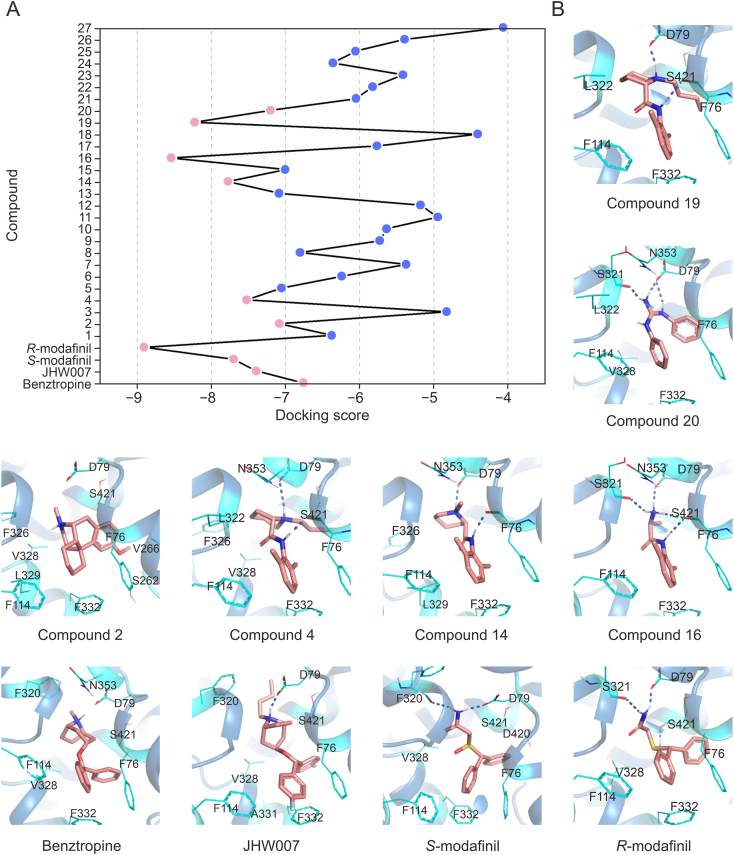
The results of induced-fit docking (IFD) of the template compounds and active compounds into the S3 site of human dopamine transporter (hDAT). (A) Distribution of docking scores for the four template molecules and the 27 compounds. (B) Docking poses of the four template molecules and the six manually selected compounds in S3 site. hDAT is shown in sky blue, the residues surrounding the pocket are in cyan, the ligand molecules are in pink, and hydrogen bonds are represented by blue dashed lines.

To predict the binding modes and affinities of candidate drugs toward hDAT, 31 compounds, including the four known atypical inhibitors (templates) and 27 candidates identified via scaffold-hopping similarity searches, were docked into the hDAT S3 binding pocket using the IFD approach. For the docking analysis, we employed the inward-open conformation of hDAT previously characterized through GaMD simulations [7]. This structural model exhibited only minor deviations compared with the recently resolved cryo-EM structure of inward-open hDAT (Protein Data Bank (PDB) ID: 8Y2E) [8]. Docking results indicated scores of −6.76 kcal/mol for benztropine, −7.39 kcal/mol for JHW007, −7.70 kcal/mol for *S*-modafinil, and −8.91 kcal/mol for *R*-modafinil. Docking scores for the 27 repurposed drug candidates are illustrated in Fig. 4A. Specifically, 12 compounds yielded docking scores above −6 kcal/mol, six scored between −7 and −6 kcal/mol, and nine displayed scores below −7 kcal/mol (Table S2). Based on docking scores and an expert evaluation of the predicted binding modes, six compounds (2, 4, 14, 16, 19, and 20) were selected for further experimental evaluation. Docking poses of these six candidates, depicted in Fig. 4B, showed consistent molecular orientations relative to the template molecules. In these complexes, the charged termini of the ligands formed stable hydrogen-bond interactions at the orthosteric pocket residues, involving Phe76, Asp79, and Ser321, while the hydrophobic termini extended towards the intracellular vestibule, engaging predominantly in hydrophobic interactions with residues Phe76, Phe114, and Phe332.

### 3.3 The reuptake function of hDAT was inhibited by the repurposed drugs

**Fig. 5:**
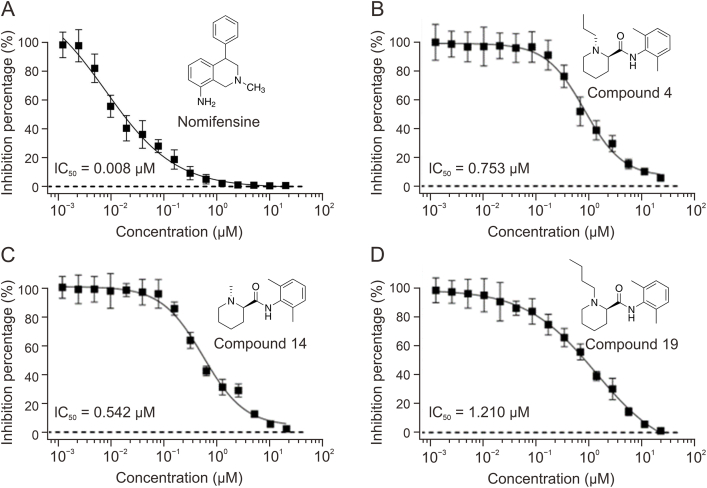
Curve of the compounds' inhibitory effects on human dopamine transporter (hDAT) using the Neurotransmitter Transporter Uptake Assay Kit. (A) Inhibition assay of nomifensine. The efficiency of monoamine reuptake begins to decline sharply at very low concentrations, with a half maximal inhibitory concentration (IC_50_) of 0.008 μM. (B–D) Translocation potency decreased with increasing concentrations of compound 4 (B), compound 14 (C), and compound 19 (D), whose half inhibitory concentrations were 0.753, 0.542, and 1.210 μM, respectively.

The DA reuptake inhibitory activities of the six repurposed drugs were evaluated using the Neurotransmitter Transporter Uptake Assay Kit through dose-response assays. This assay employs a novel fluorescent indicator dye designed to mimic neurotransmitters, along with a masking dye to quench extracellular fluorescence, enabling precise measurement of transporter-mediated uptake activity [71]. HEK293T cells stably transfected to express hDAT were utilized, with transporter expression levels confirmed via quantitative real-time polymerase chain reaction (qPCR). Results indicated that compounds 4, 14, and 19 effectively inhibited DA reuptake, demonstrating IC_50_ values of 0.753 μM, 0.542 μM, and 1.210 μM, respectively (Fig. 5). Additionally, nomifensine, utilized as a control, demonstrated potent inhibitory activity with an IC_50_ value of 0.008 μM, aligning closely with previously reported values and thereby validating the assay methodology used in this study [72].

As depicted in Fig. 5, the three active compounds (4, 14, and 19) share the same chemical scaffold (*N*-phenylpiperidine-2-carboxamide). Notably, variations in the length of the hydrocarbon chain attached to the nitrogen atom of the piperidine group appeared to influence inhibitory activity. Specifically, compounds 14, 4, and 19 exhibited increasingly longer hydrocarbon chains, correlating with progressively decreased inhibitory activity. This observation suggests that extending the hydrocarbon chain length at this position negatively affects ligand binding to hDAT. Further docking analysis (Fig. 4B) revealed that the extended hydrocarbon chains in compounds 4 and 19 did not insert deeply into the orthosteric pocket like JHW007 but instead caused significant steric hindrance near the hinge region between transmembrane helices TM1 and TM6, negatively impacting ligand binding to hDAT.

### 3.4 MOA of the identified active compounds as potential atypical inhibitors

**Fig. 6:**
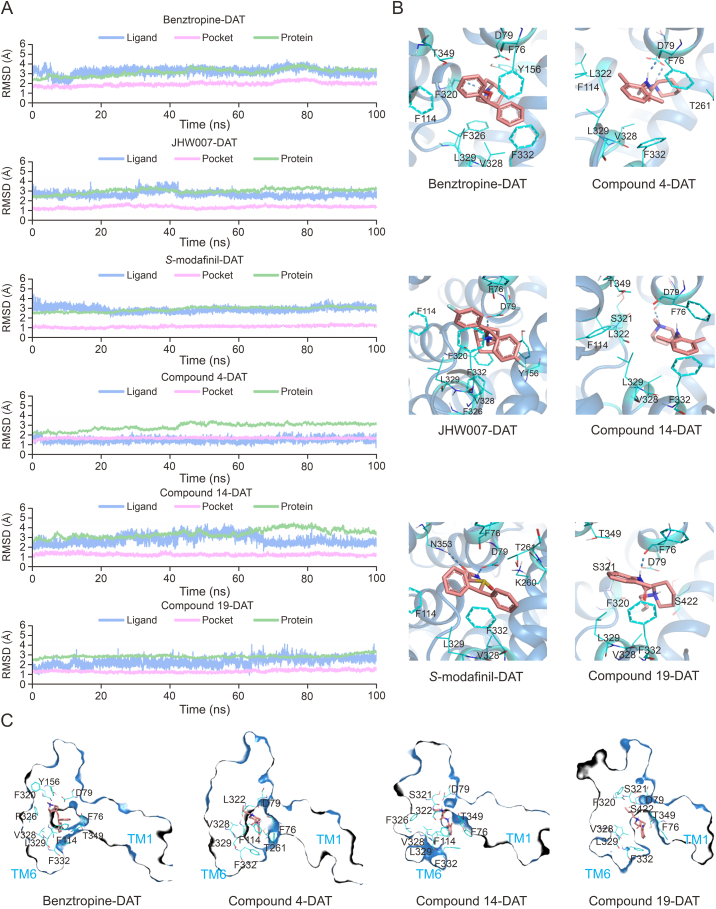
Verification of the mechanism of action (MOA) of atypical inhibitors against human dopamine transporter (hDAT) in inward-open conformation by comprehensive molecular dynamic (MD) simulations. (A) The dynamic changes of the three template molecules and three active compounds during the sampling process. (B) The representative conformations of the three template molecules and three active compounds throughout the sampling process. hDAT is shown in sky blue, the residues surrounding the pocket are in cyan, the ligand molecules are in pink, and hydrogen bonds are represented by blue dashed lines. (C) Surface representation of the structures for Benztropine and compounds 4, 14, and 19 bind to hDAT characterized through MD simulations. All of the ligands bind to the S3 site within the intracellular channel formed by transmembrane helices TM1 and TM6. RMSD: root mean square deviation.

To understand the MOA of the identified three active compounds as potential atypical inhibitors, comprehensive MD simulations were conducted to verify the interactions between hDAT and drug molecules [73,74], including reference inhibitors (Benztropine, JHW007, and *S*-Modafinil), as well as the active compounds 4, 14, and 19. Each molecule underwent three parallel 100 ns all-atom simulations (Figs. S1–S6). Analysis of the root-mean-square deviation (RMSD) of protein backbone atoms, pocket residues, and ligand heavy atoms indicated minimal fluctuations (<3 Å), confirming the structural stability of both ligands and protein throughout the simulations (Fig. 6A). To quantitatively estimate ligand binding affinities, binding free energies were calculated for the six complexes using the MM/GBSA method [75]. As shown in Table 1, the calculated binding free energies of the three newly identified active compounds were slightly weaker than those of the template inhibitors; however, all binding energies remained below the −25 kcal/mol threshold. Furthermore, the predicted binding free energies correlated well with the previously reported IC_50_ values of benztropine (312 ± 1.1 nM) [76], JHW007 (24.6 ± 1.97 nM) [77], and modafinil (6.66 ± 1.44 μM) [78] against hDAT.

**Table 1:** Estimated molecular mechanics/generalized born surface area (MM/GBSA) binding energies (Δ*G*_MM/GBSA_, kcal/mol) between molecules and human dopamine transporter (hDAT).

System	Δ*E*_ele_^a^ (kcal/mol)	Δ*E*_vdW_^b^ (kcal/mol)	Δ*G*_pol_^c^ (kcal/mol)	Δ*G*_nonpol_^d^ (kcal/mol)	Δ*G*_MM/GBSA_^e^ (kcal/mol)	IC_50_^f^ (nM)
Benztropine-hDAT	−115.81	−41.92	129.01	−5.39	−34.12 ± 2.88	312
JHW007-hDAT	−126.38	−54.98	141.36	−7.04	−47.03 ± 2.68	24.6
*S*-modafinil-hDAT	−25.52	−36.22	37.51	−5.47	−29.69 ± 3.26	6660
Compound 4-hDAT	−111.41	−40.39	129.99	−5.44	−27.25 ± 3.34	753
Compound 14-hDAT	−129.14	−35.08	140.97	−4.46	−27.71 ± 3.43	542
Compound 19-hDAT	−99.11	−38.22	115.75	−5.27	−26.84 ± 2.70	1210

IC_50_: half maximal inhibitory concentration.

a Electrostatic interaction energy (Δ*E*_ele_) in the gas phase.

b van der Waals interaction energy (Δ*E*_vdW_) in gas phase.

c Free energy of polar solvation (Δ*G*_pol_).

d Free energy of nonpolar solvation (Δ*G*_nonpol_).

e Total binding free energy, Δ*G*_MM/GBSA_ = Δ*E*_ele_ + Δ*E*_vdW_ + Δ*G*_pol_ + Δ*G*_nonpol_.

f The inhibition activity against dopamine reuptake and the concentration-gradient assay resulted in about 50% of maximum uptake inhibition by the reduction of relative fluorescence unit (RFU).

To further clarify ligand-protein interaction mechanisms at the atomic level, MIF analyses were performed on trajectory snapshots from MD simulations using ProLIF software [63] (Figs. S7–S12). Representative structural frames were extracted and visualized (Fig. 6B). Trajectory analysis revealed a consistent binding pattern wherein the hydrophobic termini of the ligands oriented toward the intracellular hydrated channel, whereas the charged protonated termini were anchored firmly at the orthosteric pocket, predominantly interacting with Asp79. Residues Asp79 and Phe76 maintained stable interactions, including hydrogen bonding, van der Waals forces, and hydrophobic interactions, throughout the simulation. Asp79 had a strong cationic interaction with the protonated end of the ligands, and Phe76 provided a stable hydrophobic interaction. This determined the orientation of the molecule towards the substrate pocket during binding. Moreover, hydrophobic interactions with residues Phe114, Val328, Leu329, Phe332, and Thr349 further stabilized ligand binding. The hydrophobic side chains of Phe76, Phe114, Leu329, and Phe332 collectively formed a steric gate, preventing ligand release into the intracellular space. Particularly, the aromatic side chains of Phe114 and Phe332 provided substantial steric hindrance and pi-pi stacking interactions, enhancing ligand retention within the binding pocket. A comparison between our modeled Benztropine-hDAT complex and the recently reported cryo-EM structure (PDB ID: 8Y2E) revealed a low average RMSD of 1.54 Å for protein complexes and 3.92 Å for ligands (Fig. S13). The higher ligand RMSD was mainly due to the release of sodium at site 2 in the cryo-EM structure, allowing the phenyl side chain of Benztropine to occupy this space. However, the orientation of Benztropine was similar in both structures, confirming the accuracy of the structure we characterized.

By comparing the binding poses of Benztropine in the cryo-EM structure 8Y2E with those of the three compounds we characterized, we observed that in the inward-open conformation of hDAT, TM1 of hDAT was opened to form a channel leading to the intracellular side connected to the orthosteric site. However, the side chains of Phe76, Phe114, and Phe332 came close to each other, obstructing the release of the ligand (Fig. 6C). This was particularly evident in the Benztropine-DAT and compound 4-DAT systems. One end of the ligand is anchored by Asp 79 at the orthosteric site, while the other end is blocked by Phe 76, Phe 114, and Phe 332 from entering the intracellular space. The ligand was trapped in the S3 pocket of the intracellular water channel, affecting the conformational changes of TM6 and TM1, thus hindering the normal substrate transport process of hDAT.

## 4 Conclusions

In this study, we developed an integrated workflow combining scaffold hopping, IFD, and experimental validation to facilitate the repurposing of drugs targeting hDAT. Benztropine-like inhibitors (benztropine, JHW007, and *R*/*S*-modafinil) were selected as reference templates for similarity searching. Utilizing WHALES descriptors to characterize global and local structural features as well as electrostatic properties, we identified 27 candidate compounds from a library of 4921 marketed or clinically developed drugs via similarity search. Subsequent evaluations using IFD and *in vitro* cellular uptake assays resulted in the identification of three active compounds (4, 14, and 19) with notable inhibitory effects on hDAT-mediated DA reuptake, exhibiting IC_50_ values of 0.753, 0.542, and 1.210 μM, respectively. MD simulations were then conducted to elucidate the binding mechanisms of these three active compounds, confirming their interactions as atypical inhibitors of hDAT. At the S3 binding site, residues Phe76 and Asp79 were identified as crucial for stabilizing atypical inhibitor interactions. Furthermore, residues Phe76, Phe114, Leu329, and Phe332 formed a hydrophobic barrier within the intracellular channel, restricting ligand release and thereby enhancing the inhibitory potency of these molecules. In summary, this study not only identified three novel hDAT inhibitors possessing unique chemical scaffolds but also demonstrated the effectiveness and broader applicability of our integrated repurposing strategy, providing valuable insights for future drug discovery efforts targeting hDAT and potentially other therapeutic targets.

## CRediT authorship contribution statement

**Ding Luo:** Writing – original draft, Validation, Formal analysis. **Zhou Sha:** Validation, Formal analysis. **Junli Mao:** Investigation, Data curation. **Jialing Liu:** Investigation, Data curation. **Yue Zhou:** Investigation, Data curation. **Haibo Wu:** Supervision, Resources. **Weiwei Xue:** Writing – review & editing, Supervision, Conceptualization.

## Declaration of competing interest

The authors declare that there are no conflicts of interest.
