# Supplementary material for: Repurposing drugs for the human dopamine transporter through WHALES descriptors-based virtual screening and bioactivity evaluation
Source: J Pharm Anal. 2025 Jun 14;15(8):101368. doi: 10.1016/j.jpha.2025.101368 (PMC12398837; doi:10.1016/j.jpha.2025.101368)
Supplement: Multimedia component 1 [file mmc1.docx]

Supplementary data for

**Repurposing drugs for the human dopamine transporter through WHALES descriptors-based virtual screening and bioactivity evaluation**

**Table S1** Property values of the 27 compounds predicted by ADMETlab 3.0.

| Index | MW (Da) | nHA | nHD | logP | nRot | LogD |
| --- | --- | --- | --- | --- | --- | --- |
| 1 | 275.15 | 4 | 1 | 1.37 | 4 | 0.99 |
| 2 | 271.19 | 2 | 0 | 3.17 | 1 | 2.83 |
| 3 | 203.13 | 2 | 0 | 2.47 | 4 | 2.56 |
| 4 | 274.2 | 3 | 1 | 2.66 | 5 | 2.38 |
| 5 | 234.17 | 3 | 1 | 2.45 | 6 | 2.45 |
| 6 | 276.17 | 3 | 1 | 3.31 | 4 | 3.30 |
| 7 | 328.24 | 2 | 0 | 4.06 | 7 | 3.48 |
| 8 | 212.08 | 2 | 0 | 3.72 | 4 | 3.47 |
| 9 | 148.09 | 1 | 0 | 2.83 | 2 | 2.55 |
| 10 | 324.11 | 6 | 2 | 2.46 | 6 | 1.60 |
| 11 | 193.11 | 3 | 2 | 2.96 | 5 | 2.78 |
| 12 | 194.09 | 3 | 1 | 3.42 | 5 | 3.08 |
| 13 | 236.09 | 3 | 2 | 2.36 | 1 | 2.38 |
| 14 | 246.17 | 3 | 1 | 1.95 | 3 | 1.74 |
| 15 | 255.17 | 3 | 0 | 3.05 | 6 | 2.94 |
| 16 | 192.13 | 3 | 3 | 0.74 | 3 | 0.79 |
| 17 | 250.16 | 3 | 1 | 3.82 | 6 | 2.82 |
| 18 | 144.12 | 2 | 1 | 2.49 | 5 | 2.19 |
| 19 | 288.22 | 3 | 1 | 3.06 | 6 | 2.74 |
| 20 | 211.11 | 3 | 3 | 1.63 | 3 | 1.84 |
| 21 | 283.12 | 4 | 1 | 2.31 | 6 | 2.07 |
| 22 | 234.13 | 3 | 1 | 2.46 | 3 | 2.67 |
| 23 | 164.08 | 2 | 1 | 2.29 | 4 | 1.89 |
| 24 | 220.16 | 3 | 2 | 2.15 | 6 | 2.34 |
| 25 | 278.1 | 3 | 1 | 1.74 | 2 | 2.62 |
| 26 | 122.04 | 2 | 1 | 1.84 | 1 | 1.45 |
| 27 | 186.16 | 2 | 1 | 3.36 | 8 | 2.74 |

MW: molecular weight; nHA: the number of hydrogen bond acceptors; nHD: the number of hydrogen bond donors; logP: the octanol-water partition coefficient; nRot: the number of rotatable bonds; LogD: the logarithm of the n-octanol/water distribution coefficient at pH=7.4.

**Table S2** The 27 compounds identified through scaffold-based similarity searching and their induced-fit docking scores with human dopamine transporter (hDAT).

| Index | Name | Docking Score |
| --- | --- | --- |
| 1 | Homatropine Bromide | -6.375 |
| 2 | Dextromethorphan | -7.082 |
| 3 | Crotamiton | -4.827 |
| 4 | Ropivacaine | -7.522 |
| 5 | Lidocaine | -7.053 |
| 6 | Cyclandelate | -6.240 |
| 7 | Retinyl acetate | -5.372 |
| 8 | Benzyl benzoate | -6.802 |
| 9 | Trans-Anethole | -5.727 |
| 10 | Acetohexamide | -5.636 |
| 11 | Butamben | -4.946 |
| 12 | Butylparaben | -5.178 |
| 13 | Carbamazepine | -7.086 |
| 14 | Mepivacaine hydrochloride | -7.774 |
| 15 | Tripelennamine hydrochloride | -7.004 |
| 16 | Tocainide | -8.540 |
| 17 | Gemfibrozil | -5.766 |
| 18 | Valproic acid sodium salt | -4.406 |
| 19 | Levobupivacaine | -8.222 |
| 20 | N, N'-Diphenylguanidine | -7.200 |
| 21 | Reparixin | -6.047 |
| 22 | Stiripentol | -5.824 |
| 23 | 4-Phenylbutyric acid | -5.416 |
| 24 | Prilocaine | -6.361 |
| 25 | Sodium gualenate | -6.055 |
| 26 | Sodium benzoate | -5.393 |
| 27 | Arundic Acid | -4.065 |
| Ref. | Benztropine | -6.758 |
| Ref. | JHW007 | -7.393 |
| Ref. | S-Modafinil | -7.699 |
| Ref. | R-Modafinil | -8.909 |

**Fig. S1.** The average root-mean-square deviations (RMSDs) fluctuations during the three parallel 100 ns simulations of the Benztropine-DAT complex, with Benztropine bound at the S3 site of human dopamine transporter.

**Fig. S2.** The average root-mean-square deviations (RMSDs) fluctuations during the three parallel 100 ns simulations of the JHW007-DAT complex, with JHW007 bound at the S3 site of human dopamine transporter.

**Fig. S3.** The average root-mean-square deviations (RMSDs) fluctuations during the three parallel 100 ns simulations of the S-Modafinil-DAT complex, with S-Modafinil bound at the S3 site of human dopamine transporter.

**Fig. S4.** The average root-mean-square deviations (RMSDs) fluctuations during the three parallel 100 ns simulations of the Compound 4-DAT complex, with Compound 4 bound at the S3 site of human dopamine transporter.

**Fig. S5.** The average root-mean-square deviations (RMSDs) fluctuations during the three parallel 100 ns simulations of the Compound 14-DAT complex, with Compound 14 bound at the S3 site of human dopamine transporter.

**Fig. S6.** The average root-mean-square deviations (RMSDs) fluctuations during the three parallel 100 ns simulations of the Compound 19-DAT complex, with Compound 19 bound at the S3 site of human dopamine transporter.


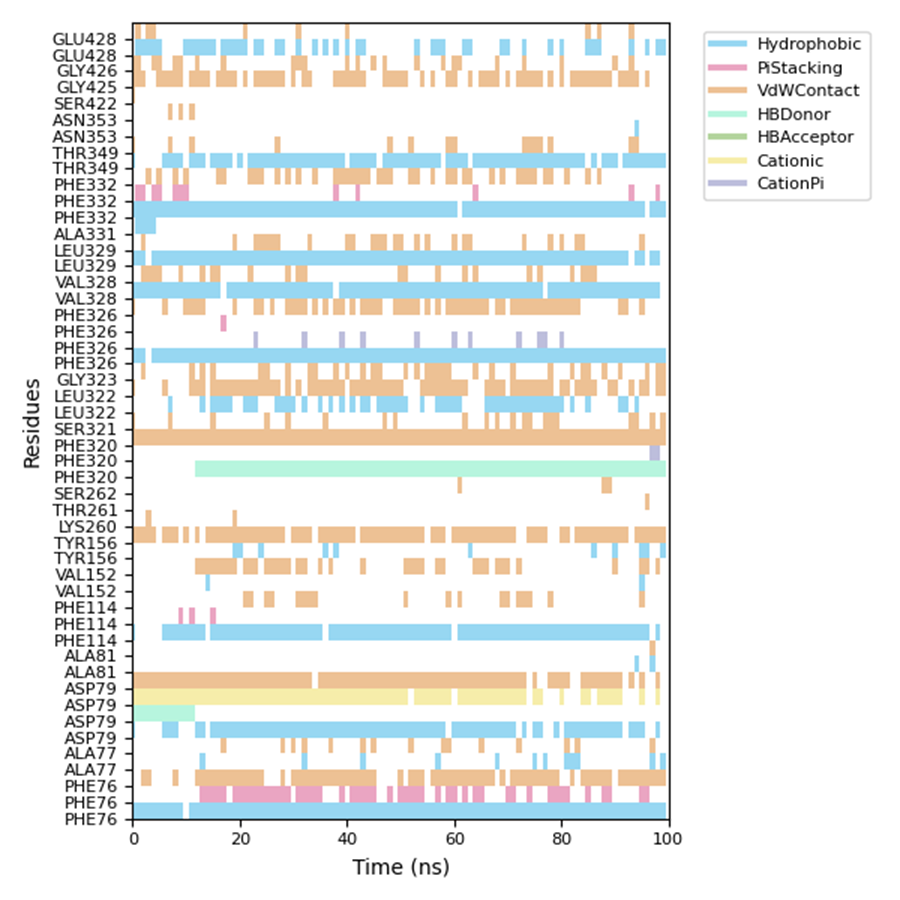


**Fig. S7.** The dynamic changes in various interactions between the ligand and the protein throughout the 100-ns simulation trajectory of the Benztropine-DAT complex.


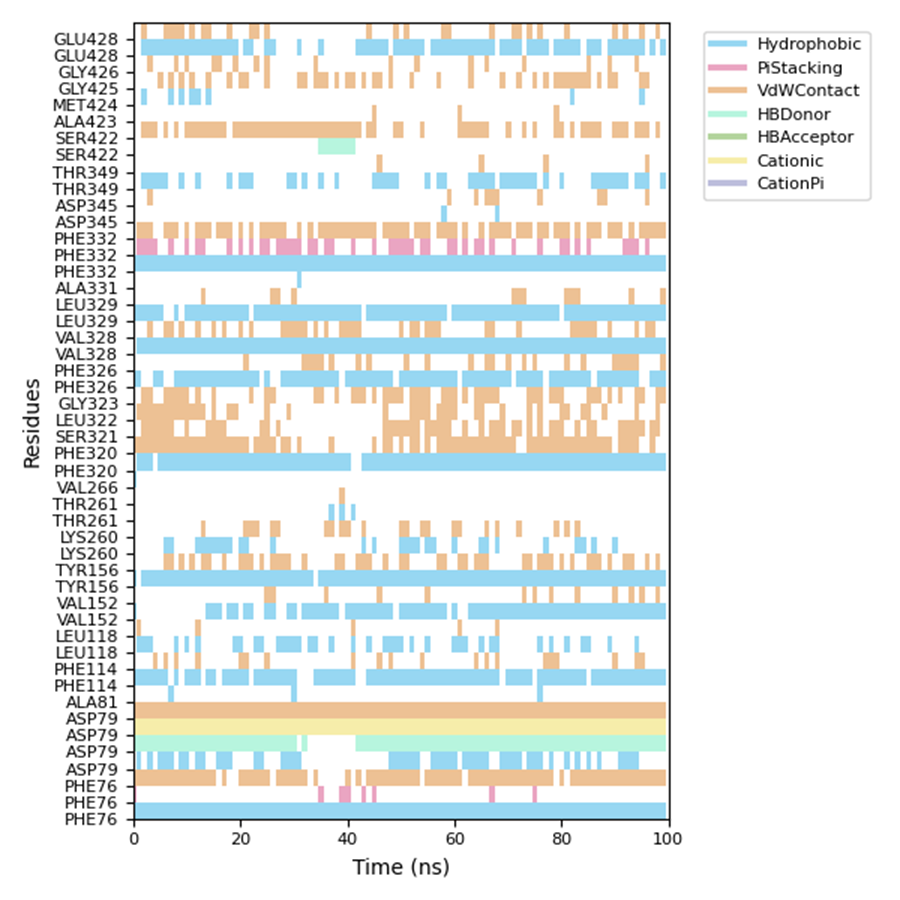


**Fig. S8.** The dynamic changes in various interactions between the ligand and the protein throughout the 100-ns simulation trajectory of the JHW007-DAT complex.


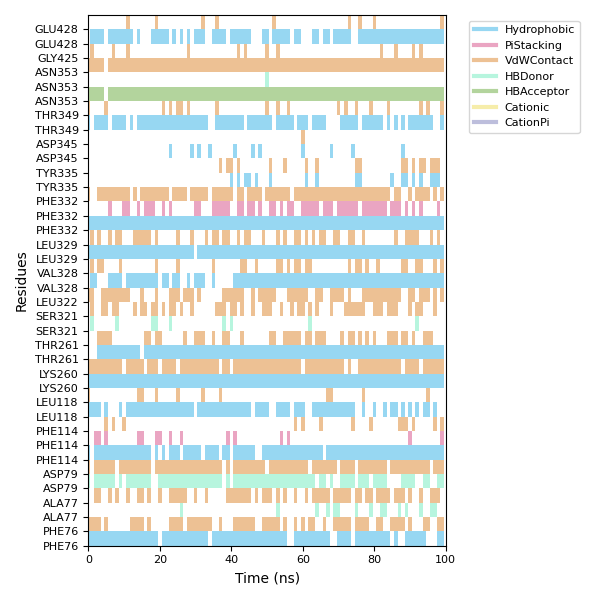


**Fig. S9.** The dynamic changes in various interactions between the ligand and the protein throughout the 100-ns simulation trajectory of the S-Modafinil-DAT complex.


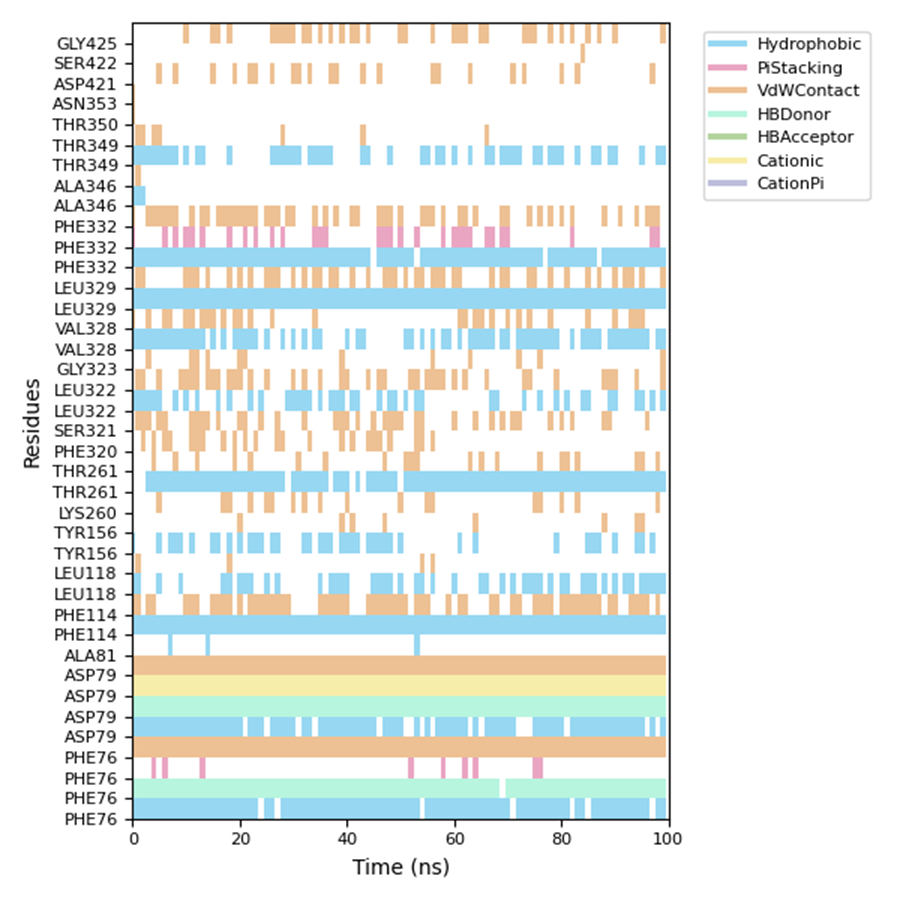


**Fig. S10.** The dynamic changes in various interactions between the ligand and the protein throughout the 100-ns simulation trajectory of the Compound 4-DAT complex.


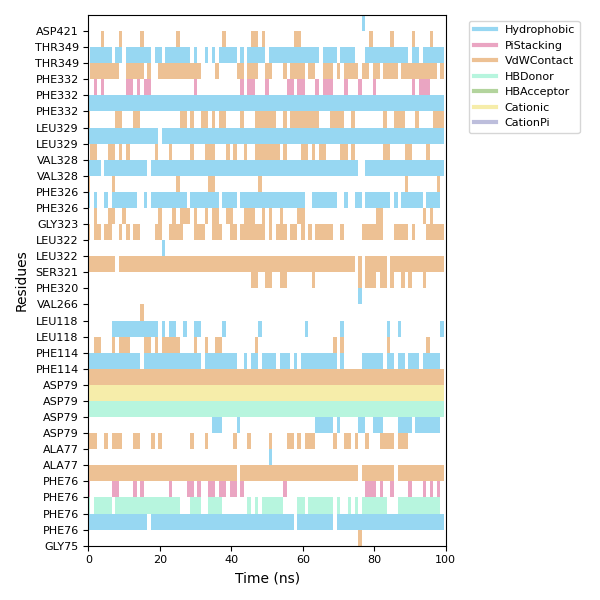


**Fig. S11.** The dynamic changes in various interactions between the ligand and the protein throughout the 100-ns simulation trajectory of the Compound 14-DAT complex.


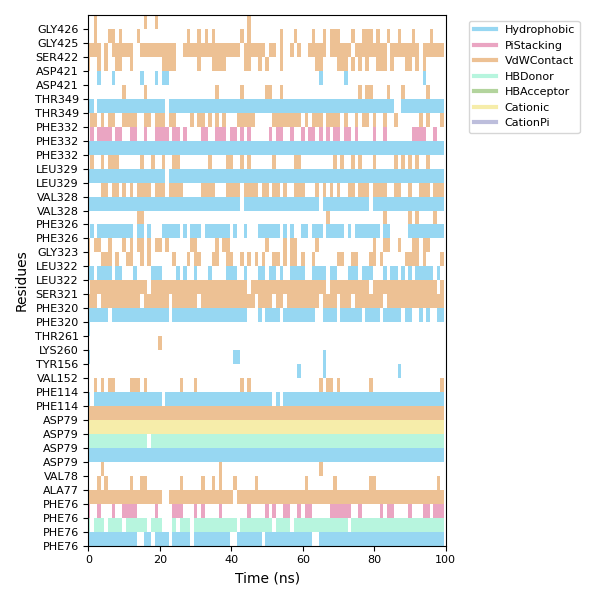


**Fig. S12.** The dynamic changes in various interactions between the ligand and the protein throughout the 100-ns simulation trajectory of the Compound 19-DAT complex.


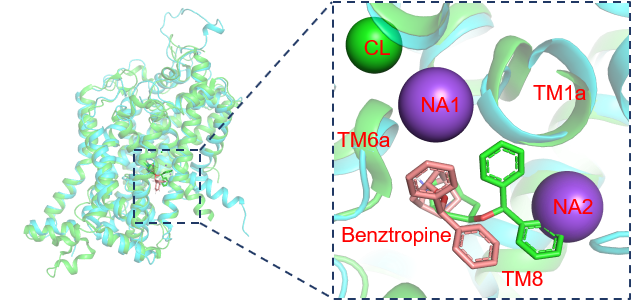


**Fig. S13.** The comparison between the Benztropine-DAT structure (blue) and the 8Y2E structure (green). Benztropine in the Benztropine-DAT structure is represented as pink sticks, and in the 8Y2E structure shown as green sticks. Two sodium ions and one chloride ion in the Benztropine-DAT structure are displayed as spheres.
